# A Natural Carbon Encapsulated in Gellan-Based Hydrogel Particles Designed for Environmental Challenges

**DOI:** 10.3390/gels10110713

**Published:** 2024-11-05

**Authors:** Camelia-Elena Tincu (Iurciuc), Mihaela Hamcerencu, Marius Sebastian Secula, Corneliu Sergiu Stan, Cristina Albu, Marcel Popa, Irina Volf

**Affiliations:** 1 Faculty of Chemical Engineering and Environmental Protection, “Gheorghe Asachi” Technical University of Iasi, 73, Prof. Dr. Docent D. Mangeron Street, 700050 Iasi, Romania; camelia_tincu83@yahoo.com (C.-E.T.); marius-sebastian.secula@academic.tuiasi.ro (M.S.S.); corneliu-sergiu.stanc@academic.tuiasi.ro (C.S.S.); cristina.albu@student.tuiasi.ro (C.A.); marpopa2001@yahoo.fr (M.P.); 2 Laboratoire de Photochimie et Ingénierie Macromoléculaires—Ecole Nationale Supérieure de Chimie de Mulhouse, Université de Haute Alsace, 3 rue Alfred Werner, 68093 Mulhouse Cedex, France; mhamcerencu@yahoo.com; 3 Academy of Romanian Scientists, Splaiul Independentei Street No. 54, 050085 Bucharest, Romania

**Keywords:** hydrogel, gellan particles, Fe^3+^ cross-linking agent, carbonaceous structures, composites, adsorption, pollutant

## Abstract

This article reports the obtention of a new gellan-based hydrogel linked with Fe^3+^ and loaded with a natural micro/nanostructured carbon designed as a contaminant’s removal from wastewater. Hydrogels are known for their water-retaining properties, high binding capacity, and eco-friendly features. The new material is expected to behave as one cost-effective and efficient sorbent, including natural carbon structures with various functional groups. The encapsulation efficiency ranges between 89% and 95%. The obtained hydrogel particles were characterized using FT-IR spectroscopy and scanning electron microscopy techniques. The hydrogel particles’ water stability was evaluated by measuring the transmittance for 10 days, and the capacity to retain water was assessed by determining the swelling degree (Q%). The results showed that hydrogel particles are stable (the transmittance value is higher than 97.8% after 10 days), and their properties are influenced by the cross-linking degree, the amount of the carbon particles encapsulated, and the concentration of gellan. For example, the Q% values and encapsulation efficiency increased when the cross-linking degree, the carbon microstructure quantity, and the gellan concentration decreased. The new hybrid material can retain Pb(II) ions and diclofenac molecules, and could be used in different adsorption–desorption cycles.

## 1. Introduction

Hydrogels play a crucial role in various applications due to their ability to retain water, adjustable properties, high capacity to bind, selectivity, reusability, and environmentally friendly nature. Hydrogels are composed of a three-dimensional structure that can absorb a significant amount of water and swell in contact with water due to their hydrophilic groups such as -NH_2_, -COOH, -OH, -CONH_2_, -CONH, and -SO_3_H. These cross-linked polymer chains form a network structure, enabling them to absorb relatively significant amounts of fluid [1].

Customizable to meet specific needs for different uses [2,3], hydrogels can be modified with particular ligands or functional groups to adsorb and retain efficiently specific contaminants from wastewater [2,4,5]. Furthermore, they can regulate the kinetics and thermodynamics of the adsorption process, enhancing the efficiency of pollutant removal [3,6,7]. Hydrogels can also complement other wastewater treatment methods like coagulation, precipitation, and membrane filtration to enhance pollutant removal efficiency [8].

Hydrogels based on natural or synthetic polymers have been used in environmental remediation. Each hydrogel has specific features and applications. A biopolymer derived from chitin, chitosan is often used to create hydrogels due to its abundant amino and hydroxyl groups, which can be complexed with metal ions. Several studies have explored chitosan-based hydrogels for metal ion removal [9]. Biopolymers derived from seaweed, alginate, and alginate-based hydrogels have been used to remove pollutants from wastewater [10]. Poly(acrylic acid) (PAA) hydrogels are widely used due to their exceptional ability to absorb water, making them highly effective [11,12]. Polyvinyl alcohol (PVA)-based hydrogels and polyacrylamide hydrogels are also known for their high water-absorbing capacity and have been studied mainly for environmental applications [2,13,14]. Gellan hydrogels are 3D networks of water-absorbing polymers made using gellan gum, a naturally derived polysaccharide. These hydrogels have gained attention for their use in various environmental applications [15]: pollutant removal from wastewater [16,17,18]; reducing soil erosion, improving soil structure, and increasing water retention [19]; creating controlled-release systems for agrochemicals [20,21]; encapsulating hazardous materials or immobilizing contaminants in soil and groundwater [22].

Gellan hydrogels are biodegradable, biocompatible, and can be functionalized to make them suitable for various environmental applications. The concentration of the polymer solution, temperature, and the presence of monovalent or divalent cations in the solution influence gellan gelling. At low temperature, the solution viscosity decreases significantly due to a single polysaccharide chain. The transition temperature from sol to gel phase transformation can range between 30 and 50 °C. However, gellan gel is formed below this temperature. The gelling process involves the formation of double helical junction zones, followed by the aggregation of double helical segments to create a three-dimensional network through complexation with cations and hydrogen bonds with water. Ordered structures remain highly stable at elevated temperatures, as divalent cations are progressively introduced. Consequently, divalent ions exhibit greater efficacy compared to monovalent ones. However, their effectiveness depends on multiple factors, such as the type of gellan gum used, the gelation process, and the environmental conditions. Research and development efforts are ongoing to optimize gellan hydrogels for environmental purposes, and they can potentially mitigate environmental challenges related to water quality, soil erosion, and pollution control [23].

Gellan hydrogels can also serve as a support matrix for immobilizing microorganisms such as bacteria or fungi, which can help biodegrade organic contaminants in wastewater. The hydrogel matrix provides a stable environment for microbial growth and activity [24].

It is important to note that the performance of gellan hydrogels in wastewater applications depends on factors such as the specific formulation of the hydrogel, the nature of the contaminants in the wastewater, and the treatment goals. Additionally, the cost and scalability of gellan hydrogel-based systems should be considered when evaluating their use in large-scale wastewater treatment processes [25,26].

Immobilizing carbon micro/nanostructures (CNMS) in hydrogels for pollutant adsorption from wastewater may offer significant advantages. This approach can enhance adsorption capacity, improve selectivity, reduce leaching, and provide sorbent physical stability [27,28,29,30].

Ferric ions (Fe^3+^) can be used as a cross-linking agent in the preparation of hydrogels for various applications [31,32]. Fe^3+^ ions can be cross-linking agents by forming coordination bonds with functional groups on the polymer chains [33], thus helping to stabilize the hydrogel structure and improving its mechanical strength and resistance in water. Hydrogels cross-linked with Fe^3+^ have proven to be highly effective in wastewater treatment applications [34] as they can efficiently capture contaminants via chelation [35]. The hydrogel properties, including porosity, pore size, and swelling behavior, can be tailored to suit specific wastewater treatment applications by adjusting the Fe^3+^ cross-linking conditions. Obtaining cross-linked hydrogels containing Fe^3+^ ions may be an effective solution for eliminating certain pollutants from the pharmaceutical sector, particularly for treating wastewater containing various medications, notably non-steroidal anti-inflammatory drugs like diclofenac. Prior research has indicated that Fe^3+^ ions can establish stable metal complexes with the drug diclofenac [36]. Consequently, Fe^3+^ ions that have not participated in the cross-linking reaction with gellan can interact with the carboxylic group present in the drug molecule, allowing the hydrogels to be utilized in wastewater treatment to eliminate pollutants from the pharmaceutical field.

Cross-linking gellan with Fe^3+^ to obtain hydrogels offers significant advantages, especially in addressing pharmaceutical pollutants. Traditional methods of hydrogel preparation often encounter challenges such as insufficient mechanical strength, limited stability, and reduced effectiveness in removing contaminants [37,38,39].

Numerous previous fabrication methods have limitations, resulting in hydrogels not possessing the structural integrity needed for efficient application in dynamic environments. Moreover, earlier methods may not have effectively utilized metal ions to improve contaminant adsorption [40,41]. Traditional hydrogels frequently fail to offer optimal pharmaceutical binding sites, decreasing removal efficiencies. Additionally, certain hydrogels are not biodegradable, which raises environmental issues after they are utilized [42].

The use of Fe^3+^ ions in gellan-based hydrogels offers significant promise, as these ions can enhance the hydrogels’ capacity to adsorb various contaminants, including pharmaceuticals. The ionic cross-linking involving Fe^3+^ ions enhances the hydrogels’ mechanical strength and enables Fe^3+^ to participate in redox reactions, potentially facilitating the degradation of certain pharmaceutical pollutants. Moreover, iron-based materials are usually more effective for environmental applications thanks to their availability and lower toxicity [16,43,44].

Hydrogels can be synthesized with specific functional groups like thiol, amino, or carboxyl groups that enhance their affinity for specific metal ions. These functionalized hydrogels are used in various adsorption applications [3]. Incorporating nanoparticles into hydrogels can enhance their adsorption capabilities. These nanoparticles can comprise materials like graphene, carbon nanotubes, or metal oxides [45].

It is important to note that the specific type of hydrogel used depends on the water contaminant and on the targeted adsorption capacity and selectivity. Researchers continue to explore new hydrogel materials to improve contaminant removal efficiency [37]. Also, organic pollutants are of interest due to their persistence in ecosystems and living organisms. Among them, a particular interest has been taken in the pharmaceutical class of contaminants [46].

The main goal of this work is to design, synthesize, and characterize gellan-based hydrogel particles cross-linked with Fe^3+^ ions and loaded with carbonaceous nano-/microstructures (CNMS). The newly developed hybrid material is used to assess its adsorption features in relation to organic persistent pollutants and heavy metal ions in multiple adsorption/desorption cycles. To the best of our knowledge, Fe^3+^ ions have not been used to create a gellan-based hydrogel. Also, the article reports an innovative development of a gellan-based hydrogel cross-linked with FeCl_3_ ions to encapsulate CNMS, having potential applications in environmental remediation.

## 2. Results and Discussion

### 2.1. Preparation of Hydrogel Microparticles Cross-Linked with FeCl_3_ × 6 H_2_O with Encapsulated CNMS 

In order to design a sustainable and feasible hydrogel, gellan, a natural biodegradable polymer, was considered to prepare the hybrid material matrix.

The gellan particles are synthesized through an ion exchange reaction between gellan in its sodium salt form and ferric chloride, shown by the following reaction:

3 Gellan-COO^−^Na^+^ + Fe^3+^Cl^−^_3_ → (Gellan-COO^−^)_3_Fe^3+^ + 3 Na^+^Cl^−^ (1)

The Fe^3+^ is a cation that tends to form complexes with ligands containing oxygen atoms, especially in negatively charged ligands like carboxylate groups.

The CNMS particles are trapped in the hydrogel mesh formed by ionic cross-linking.

The experimental program and the designated acronyms of the obtained samples are presented in Table 1.

The appearance of side complexation reactions or coordinative bonds is determined by the presence of ferric ions, which leads to the formation of a stronger gel network (with a higher cross-linking degree) than using other divalent metals of group II-A. The use of CNMS in a gellan matrix is expected to improve its mechanical properties, such as tensile strength, stiffness, and toughness. The high surface area of the carbon particles reinforces the matrix, resulting in a stronger composite material. Furthermore, incorporating activated carbon into the hydrogel network can further enhance the material mechanical strength [47,48].

### 2.2. Encapsulation Efficiency of CNMS Microparticles

**Figure 1:**
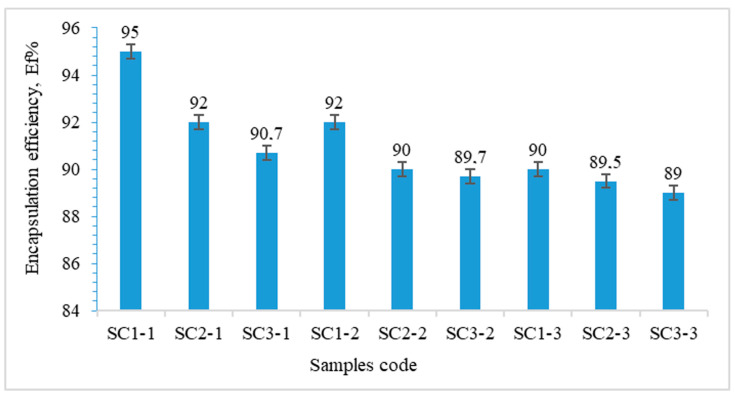
Encapsulation efficiency of CNMS particles in gellan hydrogel cross-linked with FeCl_3_.

Figure 1 shows the results regarding the immobilization efficiency of carbon particles in gellan particles.

Encapsulation efficiency depends on both the hydrogel particles cross-linking degree and the amount of CNMS particles used, as shown in Figure 1. The cross-linking degree increases when the concentration of FeCl_3_ in the cross-linking bath increases. It was observed that the immobilization efficiency decreases with the increase in the amount of encapsulated CNMS particles and when the cross-linking degree is higher. However, the overall immobilization efficiency values remained high—between 89% and 95%—for all samples analyzed.

### 2.3. FT-IR Spectroscopy Characterization

**Figure 2:**
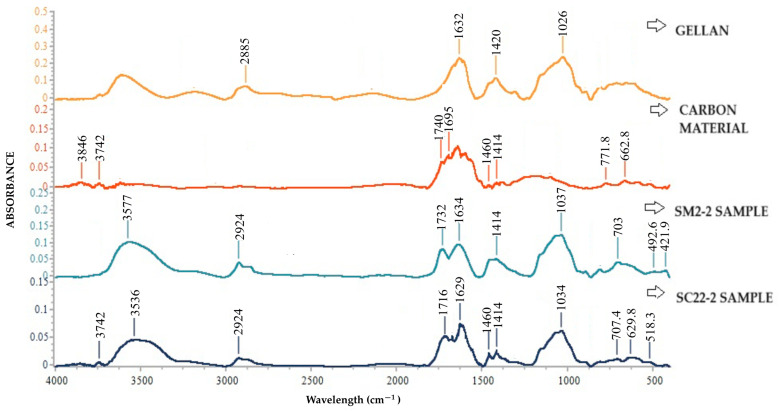
FT-IR spectroscopy for gellan, hydrogel microparticles of gellan cross-linked with Fe^3+^ (SM2-2), and for carbon particles-loaded hydrogel microparticles of gellan cross-linked with Fe^3+^ (SC22-2).

Figure 2 shows the FTIR spectra for hydrogel microparticles of gellan cross-linked with Fe^3+^ (SM2-2), carbon particles-loaded hydrogel microparticles of gellan cross-linked with Fe^3+^ (SC22-2), CNMS particles, and gellan, respectively.

Figure 2 shows that the standard gellan displays characteristic peaks around 2885, 1632, 1420, and 1026 cm^−1^. The peak at approximately 2885 cm^−1^ corresponds to the C-H vibrations. The strong band around 1026 cm^−1^ is specific to carboxylic groups from gellan and is attributed to the C-O-C stretching. The peak at 1420 cm^−1^ can be assigned to C-O stretching from the carboxylic group or the CH_3_ group from the rhamnose unit. These peaks are present in the SM2-2 and SC22-2 spectra, with only a slight variation in wavelength. Carbon particles’ absorption bands around 3700 cm^−1^ can be attributed to the stretching vibration of hydroxyl (-OH) functional groups found in phenol, alcohol, and carboxylic acid [49]. The bands at about 1740 cm^−1^ and 1695 cm^−1^ observed in the carbon particles’ sample spectrum are attributed to the C=O stretching vibrations of ketones, esters, or aliphatic acids [50]. The peaks observed at 662 cm^−1^ and 771.3 cm^−1^ correspond to the vibration of alkanes’ and alkynes’ C-H bending [51]. These carbon particle spectrum bands were assigned to stretching vibrations of C-OH groups (phenolic, ethers) from quinones, carboxylic acid, and alcohols, respectively [52].

For SM2-2 and SC22-2 FT-IR spectra, as we mentioned earlier, all the characteristic peaks from gellan are found at wavelengths that slightly changed. It could be observed that, in the spectrum of SM2-2, a peak occurs at 1732 cm^−1^. The two peaks within the gellan spectrum, located at 1632 cm^−1^ and 1420 cm^−1^, could be attributed to the asymmetric and symmetric stretching vibrations of the COO– in the gellan chains, respectively [53]. According to the literature [54], the difference in the wavelengths between the asymmetric (ν_as_(COO−)) and symmetric stretching vibrations (ν_s_(COO−)) can be used to confirm whether Fe^3+^ ions were bound to the COO− groups. If the separation between the two vibrations, ν_as-s_ = ν_as_(COO−) − ν_s_(COO−), is greater than 200 cm^−1^, it indicates the presence of unidentate binding, while a separation of approximately 200 cm^−1^ suggests bridging binding. In the case of gellan hydrogels cross-linked with Fe^3+^, it was observed that the ν_as_(COO−) was split into peaks at 1732 cm^−1^ and 1634 cm^−1^ for SM2-2 hydrogel, and 1716 cm^−1^ and 1629 cm^−1^, while the ν_s_(COO−) for the hydrogel was at 1414 cm^−1^. This resulted in two wavelength separations (Δν_as_-_s_) of 318 cm^−1^ and 220 cm^−1^ for the SM2-2 hydrogel, and 302 cm^−1^ and 215 cm^−1^, suggesting the coexistence of unidentate and bridging binding between COO− and Fe^3+^ in both hydrogels. These results could also indicate that incorporating the carbon particles within the hydrogel could prevent the cross-linking of carboxylic groups from gellan with the Fe^3+^ [53]. Also, the peak at 421.9 cm^−1^ and 492.6 cm^−1^ within the SM2-2 spectrum, and the peak at 518.3 cm^−1^ from the SC22-2 spectrum, suggest that the coordination bond of Fe ^3+^ with COO– groups within gellan occurs [55].

### 2.4. Scanning Electron Microscopy Characterization

The surface morphology of the hydrogel and composite particles was analyzed. SC2-2 and SC2-3 samples contain the same amount of encapsulated material but differ in the degree of cross-linking.

**Figure 3:**
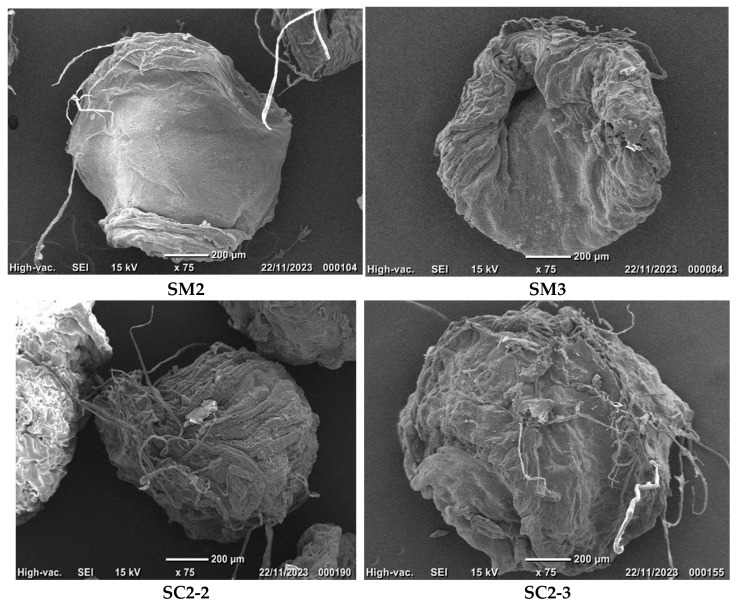
Scanning electron microscopy photographs of particles cross-linked with Fe^3+^ ions with different concentrations in the cross-linking bath 20 mM (SM2) and with 30 mM (SM3), and for the same samples that contain encapsulated carbon particles, namely SC2-2 (concentration of 20 mM Fe^3+^ in the cross-linking bath) and SC2-3 (concentration of 30 mM Fe^3+^ in the cross-linking bath).

Figure 3 displays the scanning electron microscopy micrographs obtained from the analyzed samples.

The observed particles, with and without encapsulated carbon particles, are spherical and have a diameter of approximately 1 mm. The particles without encapsulated carbon particles have a smooth surface, while the SM3 sample has a more compact surface due to a higher cross-linking degree. In the case of the micrographs for samples with encapsulated carbon particles SC2-2 and SC2-3, the hydrogel particles are also spherical with the carbon particles encapsulated. The SC2-2 composite particles show a microfibrillar structure on their surface, which is not observed for SC2-3 composite particles. This is due to a higher degree of cross-linking, resulting in a more compact hydrogel structure.

### 2.5. Stability of Gellan-Based Hydrogel Particles in Water

The stability of hydrogel particles in water, both with and without immobilized carbon particles, was tested by measuring the turbidity (transmittance, T%) of bidistilled water, in which the hydrogel particles were suspended for different periods. Less stable hydrogel particles can cause greater turbidity in the environment they are kept in due to the detachment of some polymer fragments from their surface that are not well caught in the polymer network. Similarly, the composite particles with encapsulated carbon microparticles can also result in material diffusion from hydrogel particles that are not well cross-linked and are, therefore, unstable.

Table 2 shows the transmittance values obtained at a wavelength of 550 nm for all the samples after various periods.

The stability of the hydrogel particles in aqueous media is affected by the concentration of the FeCl_3_ solution in the cross-linking bath and the amount of carbon particles, as can be observed from the transmittance values (T%) provided in Table 2. It is worth noting that the stability of all samples depends on the degree of cross-linking of hydrogel particles. Based on the samples collected, it was observed that the transmittance value decreases as the concentration of FeCl_3_ in the cross-linking bath increases, which consequently leads to a decrease in cross-linking density (SM1, SM2-2, SM3) after 10 days of being maintained in double-distilled water. Additionally, the polymer concentration affects the transmittance value, as it tends to decrease with increased polymer concentration (SM2-1, SM2-2, SM2-3). Ideally, the transmittance value should increase with the cross-linking degree, but this was not observed. The transmittance value was determined by the diffusion of small amounts of Fe^3+^ ions trapped in the hydrogel matrix that did not react with the carboxylic groups from gellan.

Previous studies have shown that it is possible to prepare alginate hydrogels by cross-linking them with FeCl_3_. It has been observed that Fe^3+^ ions bind to both glucuronic and mannuronic groups of the polymer and have a strong affinity for molecules containing carboxylic groups. However, alginate hydrogels are limited by the leakage of metal ions used for cross-linking, which can weaken the hydrogel and eventually lead to its dissolution. Compared to Ca^2+^ cross-linked hydrogels, Fe^3+^ cross-linked alginate hydrogels exhibit superior stability properties, particularly in biofluids or high ionic strength solutions, and can be used in various long-term applications. Among other metal cations, Ba^2+^ cations were reported to have minimal diffusion from the hydrogel [56]. The binding strength of Fe^3+^ to the carboxylic groups in alginate was found to be similar to that of Ba^2+^ [57]. The release of Fe^3+^ cations from Fe^3+^ cross-linked alginate particles has been studied and found to be affected by ionic strength, pH, and temperature. In an HCl solution (pH 1.2), 30% of Fe^3+^ was leached from Fe^3+^ cross-linked alginate particles within 24 h, while in PBS (pH 7.4), 15% was released [58]. At low pH, the carboxyl groups in polysaccharides are protonated, which reduces the electrostatic interaction with Fe^3+^ and leads to faster Fe^3+^ diffusion from alginate-based hydrogels [31].

Based on the results obtained from transmittance measurement, it has been observed that, in the case of gellan hydrogels, there is a possibility of diffusion of Fe^3+^ that did not participate in the formation of the coordination bonds within the polymer matrix. However, their quantity is negligible, and the transmittance value does not fall below 98%, even after 72 h of keeping gellan particles cross-linked with Fe^3+^ in water. As the cross-linking degree increases, the stability of the particles also increases. However, this also leads to increased moles of Fe^3+^ ions in the cross-linking bath that react with the carboxylic groups within gellan. With higher cross-linking, the meshes of the polymer network became smaller, and the Fe^3+^ ions that did not participate in the complexation reaction with the carboxylic groups but were absorbed from the cross-linking bath in the polymer network can diffuse from the polymer matrix over time. Gellan particles cross-linked with Fe^3+^ ions exhibit excellent water stability compared to other metal ions used for gellan cross-linking. For instance, in the case of the gellan hydrogel obtained by ionic cross-linking with Ca^2+^ ions, after 48 h of being kept in double-distilled water, the transmittance value ranged between 95.9% and 96.3% [59]. The transmittance value for gellan-based hydrogel particles cross-linked with magnesium acetate after 24 h of keeping them in bidistilled water varied between 95.6% and 98.2% [60], and for hydrogel particles cross-linked with Zn^2+^ ions, the transmittance value after 48 of hours of particle maintained in double-distilled water was between 97% and 99.2% [61].

It can be concluded that hydrogel particles made from gellan and cross-linked with FeCl_3_ remain stable in bidistilled water for extended periods. The stability of these particles is comparable to that of gellan particles cross-linked with zinc acetate. These findings suggest that gellan-based hydrogel particles cross-linked with FeCl_3_ can be used in wastewater treatment applications. The transmittance value of hydrogel particles decreases as the concentration of gellan (SM2-1, SM2-2, SM2-3) increases, and it is believed to be a consequence of unreacted Fe^3+^ ions diffusing from the polymer matrix into the water. As the concentration of gellan increases, more carboxylic groups are available to react with Fe^3+^ ions. The molecular weight of the structural unit in gellan is 646 Da, allowing us to calculate the number of moles of carboxylic groups in each concentration of gellan used to obtain hydrogel particles. The gellan solution with a concentration of 0.5% contains 7.74 × 10^−5^ moles of carboxylic groups, while the gellan solution with a concentration of 1% includes 1.55 × 10^−4^ moles of carboxylic groups. The gellan solution with a concentration of 1.5% contains 2.32 × 10^−4^ moles of carboxylic groups. For cross-linking, 25 mL of 20 mM FeCl_3_ × 6 H_2_O solution containing 2.5 × 10^−4^ moles of Fe^3+^ ions was used. One mole of Fe^3+^ can react with 3 moles of carboxyl groups. Therefore, the number of moles of Fe^3+^ ions in the cross-linking bath is in excess. However, steric hindrances between macromolecules may prevent some carboxylic groups from reacting. For example, after keeping the particles in distilled water for ten days, the transmittance value for the SM2-3 particles was equal to that obtained for the SM2-2 particles.

In the case of hydrogel particles that include carbon particles, it was found that the transmittance value remains unchanged or slightly higher even after keeping the particles in double-distilled water for 10 days. There are only two exceptions, samples SC22-1 and SC22-3, where the transmittance value is slightly lower, however, the difference is not significant enough to be considered statistically relevant. The large specific surface area of the carbon particles contributes to the strength of the gellan-based hydrogel network. By incorporating carbon particles into the hydrogel network, the overall mechanical strength of the composite material can be further improved. In this case, the transmittance value tends to decrease as the cross-linking degree of the hydrogel particles and the amount of carbon particles inside the matrix increase.

Additionally, the carbon particles may diffuse out of the polymer matrix. For instance, sample SC3-3 exhibits a transmittance value of 97.8% due to the high degree of cross-linking and large amount of immobilized carbon particles. The high degree of cross-linking causes the polymer network meshes to become smaller [62], which leads to greater rigidity in the hydrogel structure and makes it challenging to fully integrate all of the CNMS, resulting in lower encapsulation efficiency. The differences in transmittance values obtained did not show any statistical significance according to the Student T statistical test, with a *p*-value greater than 0.05. This further supports the stability of the particles in an aqueous environment.

### 2.6. Determination of the Ability of Hydrogel Particles to Retain Water

In order to determine the diffusion of aqueous solutions within the hydrogel, the swelling degree (Q%) was evaluated for all samples in an aqueous solution of NaCl 0.05 M, pH = 6.5, until equilibrium was reached (up to 1880 min). The cross-linking degree, the amount of carbon particles encapsulated, and the polymer concentration were analyzed to assess their impact on the swelling degree. A higher swelling degree indicates a higher ability of hydrogels to absorb water or aqueous solutions, making them effective in absorbing pollutants, heavy metals, organic compounds, or other contaminants from wastewater. Understanding the kinetics of swelling is crucial in determining the rate at which hydrogels absorb and release water or contaminants. This information is essential in designing efficient wastewater treatment systems using hydrogels.

**Figure 4:**
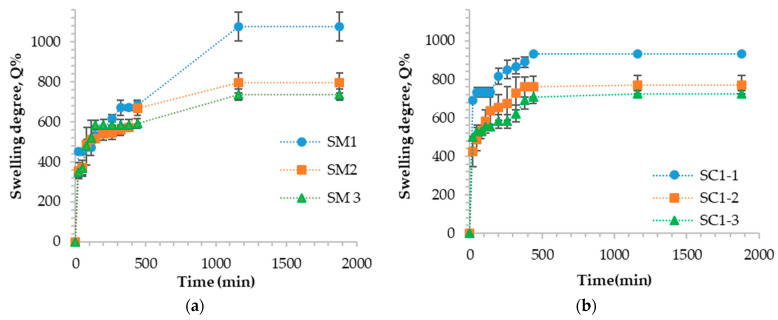

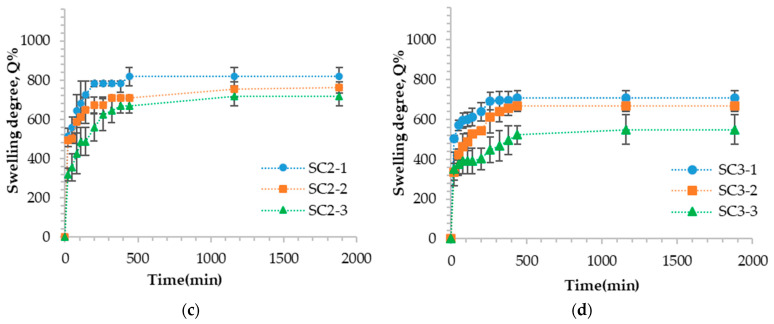
The variation of the swelling degree over time for samples with different cross-linking degrees: (**a**) SM1, SM2, SM3; (**b**) SC1-1, SC1-2, SC1-3; (**c**) SC2-1, SC2-2, SC2-3 (**d**) SC3-1, SC3-2, SC3-3.

Figure 4 shows the swelling degree over time for samples with different cross-linking degrees, with or without different amounts of encapsulated carbon particles.

The analysis of all samples shows that the swelling degree (Q%) decreases with the cross-linking degree. Samples that do not contain encapsulated carbon particles have a slightly higher swelling degree value. The swelling degree for gellan-based hydrogel particles cross-linked with Fe^3+^ ions shows similar results to those of gellan particles cross-linked with other metal ions. In previous research, the swelling degree values of gellan particles cross-linked with zinc ions with different cross-linking degrees varied between 1095.2% and 1360.1%, depending on the amount of zinc ions within the cross-linking bath [63]. For cross-linked gellan hydrogel particles with magnesium acetate, the swelling degree varied from 1536 to 1756% and depended on the particle cross-linking degree [64]. The swelling degree values of gellan particles cross-linked with Fe^3+^ ions ranged from 735.8% to 1077%. The lower swelling degree values of Fe^3+^ cross-linked hydrogel particles compared to other metal ions indicate that they are more stable and can be used in various applications.

For samples containing carbon particles encapsulated, the value of Q% also increases as the cross-linking density decreases in all analyzed cases. However, the value of Q% also depends on the amount of carbon particles encapsulated in the hydrogel particles. Therefore, the swelling degree values decrease with the increase in the amount of encapsulated carbon particles. Nevertheless, the swelling degree was maintained at optimal values for wastewater treatment applications. The Q% value for samples with 10 mg of carbon particles encapsulated (SC1-1, SC1-2, SC1-3) is similar to the Q% value of hydrogel particles without encapsulated carbon particles (SM1, SM2, SM3). However, the Q% value decreases for samples containing 30 mg of the encapsulated carbon particles, and this decrease is more pronounced as the cross-linking degree increases. For instance, the Q% value for sample SC3-1 is 707%, for SC3-2, it is 667%, and for SC3-3, it is 548.64%. Despite the encapsulation of carbon particles into the hydrogel structure, the Q% value does not decrease significantly because the composite material can also absorb water.

**Figure 5:**
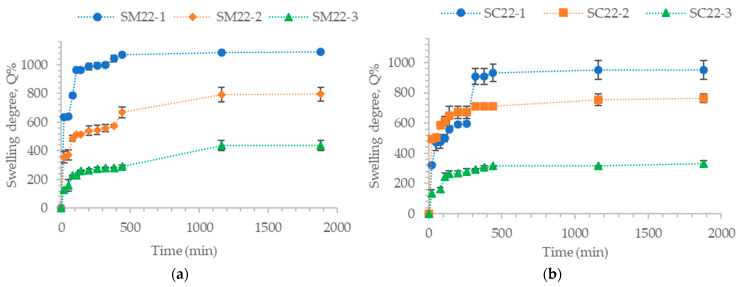
Variations in hydrogel particle swelling degree over time for samples cross-linked with Fe^3+^ containing different concentrations of gellan without carbon particles encapsulated (**a**) and with encapsulated carbon particles (**b**).

The graphs in Figure 5 illustrate the impact of different concentrations of gellan on the swelling degree in the samples, with and without encapsulated carbon particles. The concentration of the cross-linking solution and the amount of carbon particles remained constant at 20 mM and 20 mg, respectively. Only the concentration of the gellan solution was varied, with concentrations of 0.5%, 1%, and 1.5%.

Based on Figure 5, it is obvious that the swelling degree is higher in the samples without encapsulated carbon particles. Additionally, the degree of swelling decreases with polymer concentration. As previously mentioned, while discussing the stability of gellan particles cross-linked with FeCl_3_ in aqueous media, the number of functional groups (carboxylic) increases at higher gellan concentration, and the amount of moles of Fe^3+^ ions is in excess, resulting in a higher cross-linking degree. However, the number of functional groups that can interact with Fe^3+^ ions is limited due to steric hindrances between macromolecules. Thus, the degree of swelling has a lower value for hydrogel particles cross-linked with Fe^3+^ ions containing a gellan concentration of 1.5%.

The Student T-test results indicate a significant difference, with a *p*-value of less than 0.05, in the swelling degree values between sample SM1 and the samples containing encapsulated CNMS obtained at Fe^3+^ concentrations of 20 mM and 30 mM concentrations in the cross-linking bath.

**Figure 6:**
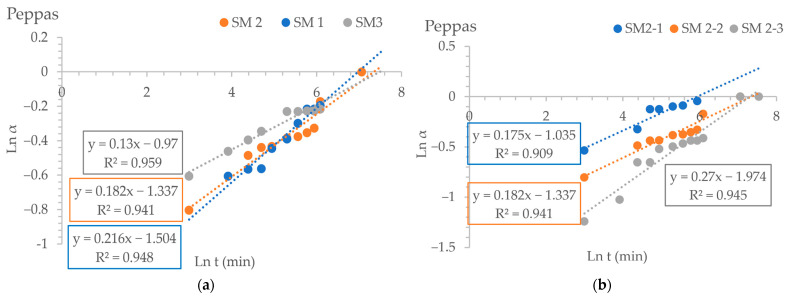

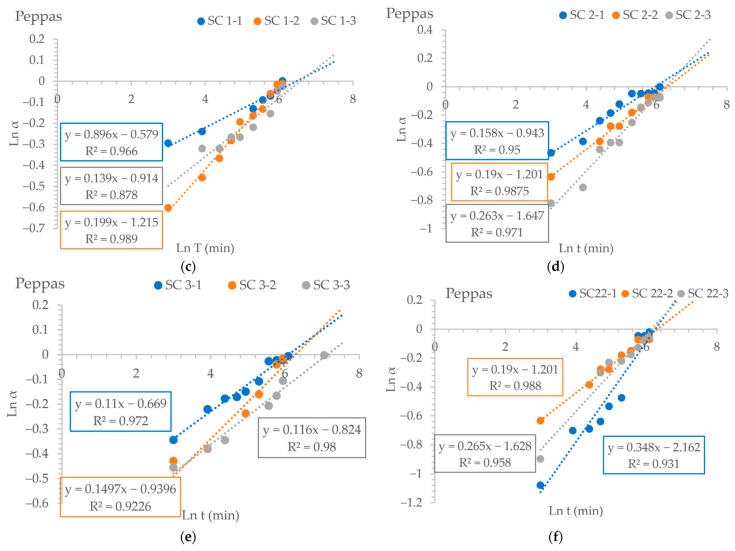
Plots of swelling kinetics using the Peppas–Korsmeyer model for samples without encapsulated CNMS where the cross-linking degree is different (**a**), for samples without CNMS differing by the amount of gellan used (**b**), for SC1 samples with various amounts of encapsulated CNMS (**c**), for SC2 samples with varying amounts of encapsulated CNMS (**d**), for SC3 samples with different quantities of encapsulated CNMS (**e**), and for samples containing 20 mg of encapsulated CNMS (**f**) that differ in the amount of polymer used in obtaining hydrogels.

Figure 6 shows the swelling analyzed using the Peppas model.

Values less than 0.5 of the exponential factor *n* indicate that the absorption mechanism of the aqueous solution is governed by diffusion. Fickian diffusion occurs when the polymer’s relaxation time (tr) is longer than the solvent’s diffusion time (td). On the contrary, non-Fickian diffusion happens when tr is approximately equal to td, and Fick’s laws of diffusion cannot explain it [65]. The diffusion of aqueous solutions through the hydrogel is influenced by the movement of water molecules within the hydrogel and the subsequent relaxation of the macromolecular chains. When the exponential factor (n) has small values, it indicates that the mechanism is Fickian. According to the literature, the high porosity of the polymer may have caused the extremely low n value (less than 0.3) in the Ritger–Peppas release kinetic model because the pores on the polymer surface allowed water to diffuse into the matrix instantly [64]. Table 3 shows the n, k, and τ values obtained after integrating the swelling data obtained in different kinetic diffusion models.

The data in Table 3 demonstrate that the swelling behavior of the gellan hydrogel, cross-linked with Fe^3+^ ions, conforms to second-order kinetics. Following the second-order kinetic model, the swelling rate is directly linked to the square of the water absorbed by the hydrogel particles until they reach equilibrium. As a result, the swelling rate decreases rapidly as time passes due to its dependence on variations in osmotic pressure. The τ value is a measure of the swelling speed, so it is a measure of the diffusion resistance of water; the smaller the τ value, the higher the swelling speed [63].

The concentration of Fe^3+^ ions in the cross-linking bath affects the value of τ, and as the degree of cross-linking of the hydrogel particles increases, the parameter τ decreases. The τ value is higher for hydrogel particles that do not contain encapsulated CNMS. This could be due to a slower diffusion rate of the aqueous solution inside these hydrogel particles, possibly because of intramolecular bonds formed between the macromolecule’s functional groups, such as hydrogen bonds. The hydrogen bonds may be cleaved gradually in time, because the pH of the swelling medium is 6.5 and, the pKa for the carboxylic groups is 3.5. After cleaving the hydrogen bonds, the hydrogel particles were able to absorb the water.

In most cases, we notice that the value of the parameter τ increases as the amount of CNMS in the samples with encapsulated CNMS increases. Therefore, the water diffusion rate decreases as the amount of CNMS in the hydrogel particles increases. The reason for this behavior is that the micro/nanostructured material is located within the pores of the polymer matrix, resulting in a reduced adsorption of aqueous solution. These findings can also be linked to the extent of swelling, which decreases as the quantity of CNMS in particles increases.

### 2.7. Pollutant Removal Assessment

In these tests, two important model pollutants were taken into account. The highly toxic Pb(II) was considered a representative heavy metal ion pollutant, whereas diclofenac molecule was chosen as an organic model pollutant due to its known high persistence in the environment. The sorption tests were performed on sample SM2, whose retention capacity of the two pollutants was compared with those of SC22 composite microparticles and non-encapsulated carbon particles.

In the case of Pb(II) ions, sorption experiments were performed in the batch system using 25 mL of Pb(II) solution under continuous stirring with 150 rpm (GFL3031) at 23 ± 1 °C, whereas for the diclofenac molecule, 100 mL batch reactors and initial concentrations of 100 mg/L and 0.4 g/L sorbent doses were used. The stirring rate values ensure that all active centers of adsorbent are available for pollutant removal. All experiments were performed in duplicate, and three absorbance readings were done for each measurement.

Lead speciation in aqueous solutions is mainly controlled by pH and redox potential. The cationic elementary Pb^2+^ form dominates the lead speciation in water for pH lower than 6 [66]. In this view, the adsorption tests were performed at pH 5, using 0.4 g/L of the adsorbent dose. The study was conducted in a range of lead concentrations usually found in industrial aqueous contaminated effluents (20 mg/L).

The composite adsorbent structure was designed as a porous one, resembling a cellular matrix that facilitates the diffusion of lead ions due to a larger contact surface and a higher number of binding sites. Also, it is known that divalent cations of transition metals such as Zn^2+^, Cu^2+^, and Pb^2+^ form stronger gels than those of metals in Group 2 [67].

The adsorption of metal ions from wastewater by gellan hydrogels occurs through several mechanisms, including physical adsorption, ion exchange, ionic bonds, and chemical complexation. The choice of the mechanism depends on the properties of the gellan hydrogel, the specific metal ions in the wastewater, and the desired treatment goals. The ability to tailor gellan hydrogels with specific functional groups and properties allows for customizing adsorption processes to target particular metal ions in wastewater [68,69,70].

The results demonstrate that all three materials selected for this test (CNMS, SM2, SC22) are effective sorbents for the uptake of contaminants from wastewater. In the case of Pb(II) ion sorption, the maximum adsorption capacities are 31.02 mg/g for SC22, 28.7 mg/g for SM2, and 14.8 mg/g for CNMS at pH 5 and 23 °C. For the Pb(II) ions, the equilibrium occurred in less than 4 h, whereas in the case of diclofenac molecule, only a quasi-equilibrium was obtained after about 24 h.

The retention of different pollutants by the characterized composite particles is due to a complex mechanism involving a physical phenomenon—sorption on the surface and within the polymer matrix—as well as the involvement of some chemical interactions (hydrogen, ionic, or even coordinative bonds).

**Figure 7:**
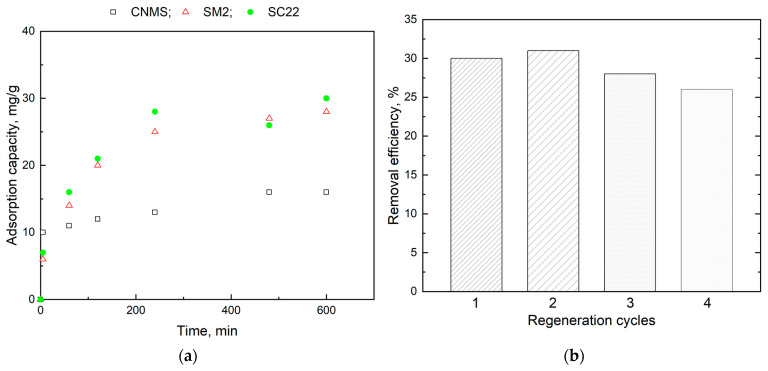
Sorption capacity in relation to Pb^2+^ ions onto CNMS, SM2, and SC22 (**a**) and regeneration cycles of SC22 (equilibrium time 600 min) (**b**).

Figure 7 shows the kinetics of the retention of Pb(II) ions, expressed in terms of sorption capacity variation in time and several regeneration cycles. Their retention capacity is lower in the case of carbon particles, and the process is mainly based on adsorption on the pore surface. A higher retention capacity is obtained in the case of composite particles, which is due to both interactions, such as hydrogen bonds with the polymer matrix, and by the possible complexation of metal ions to the free functional groups of gellan (remaining available after cross-linking by complexation with Fe^3+^ ions). Naturally, the highest retention capacity is achieved in the case of composite particles, to which, in addition to interactions such as hydrogen bonds and coordinative ones, adsorption in the pores of carbon particles adds up. For all types of analyzed particles, the equilibrium of the retention process of Pb(II) ions is reached after approximately 10 h.

Spent adsorbents were regenerated using a 3 M KCl solution. Figure 7b shows the removal efficiency achieved on SC22 after 10 h in successive desorption processes. After the primary regeneration cycle, relatively higher removal efficiency was obtained in relation to Pb(II) ions, which is most likely due to a thorough washing of the embedded CNMS in SC22. Thus, more ions can reach ion exchange sites on the surface of CNMS. Also, chloride ions could enter the microporous structure of CNMS during the desorption process.

Furthermore, the entrapped chloride ions could form complexes with Pb(II) ions during the succeeding adsorption process. However, this complexation process may be irreversible due to the formation of covalent bonds. This might partially explain the slight decrease in the adsorption capacity of Pb(II) ions during further regeneration cycles. Another reason for the decrease in the adsorption capacity in relation to Pb(II) ions could be assigned to the matrix degradation/deformation of SC22.

**Figure 8:**
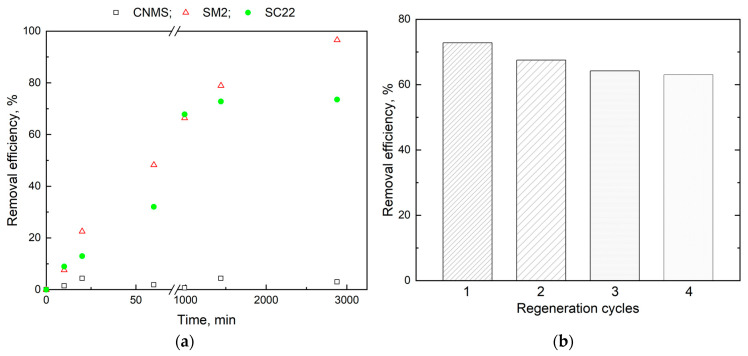
Removal efficiency of diclofenac by sorption on CNMS, SM2, and SC22 (**a**) and regeneration cycles of SC22 (equilibrium time 1440 min) (**b**).

Figure 8 shows diclofenac’s retention efficiency (%) and reutilization cycles. Although the adsorption process contributes to the drug’s retention, it is less significant than the retention of metal ions onto carbon particles. This is due to the large size of the organic molecule, which makes it difficult to diffuse into the carbon particles’ pores, compared with Pb(II) ions. Also, the pronounced hydrophobic character of the carbon particles does not favor physical interactions with the drug molecules, which exhibit a strongly polar character. Conversely, the particles without carbon entities in the composition show a high diclofenac retention capacity, given the interactions of the type of intense hydrogen bonds that appear between them (which contains carboxylic groups in the structure) and the –OH or even –COOH groups of the polymer matrix. In between are the composite particles, in which the carbon layers replace part of the polymer, reducing the number of functional groups capable of engaging in hydrogen bonds with the drug. It should be noted that the equilibrium of the drug retention process is established in this case at much longer times (around 50 h), reaching almost maximum efficiency in the case of particles without filler material.

Spent adsorbents were also regenerated using a 3 M KCl solution. Figure 8b shows the removal efficiency achieved on SC22 after 1 day in successive desorption processes. Compared to the metal ion adsorption process, the removal efficiency in relation to diclofenac molecule decreases with each regeneration cycle. This may be due to the degradation of the polymeric matrix that is not significantly balanced by the washing of CNMS, considering the relatively poor adsorption capacity of CNMS towards diclofenac molecule.

The findings indicated that hydrogel particles containing encapsulated CNMS can be filtered, regenerated, and reused for a minimum of four cycles of pollutant absorption and desorption without a notable decrease in their ability to remove lead ions and diclofenac molecules.

Table 4 shows several results reported in literature concerning lead ions and diclofenac removal, respectively, on various synthesized polymeric composites.

In case of Pb^2+^, most literature studies report adsorption tests carried out at relatively high values of initial concentration (100–2000 mg/L), which means a high driving force for the adsorption process. In the present study, the adsorption tests were conducted at relatively low values of initial concentration (20 mg/L), commonly met in natural polluted effluents. In relation to this range of initial concentration of Pb^2+^, the adsorption capacity of hydrogel particles reported herein (31.02 mg/g for SC22) is significant.

The adsorption capacity of hydrogel composites in relation to the diclofenac molecule was relatively high—195.7 mg/g.

Nevertheless, in order to evaluate accurately the adsorption capacity, in our future work the equilibrium studies will be performed.

## 3. Conclusions

Hydrogel particles were developed based on gellan that was cross-linked ionically with Fe^3+^ ions and had potential applications in wastewater treatment.

Carbon particles were encapsulated in the hydrogel matrix to improve their ability to absorb water pollutants. The encapsulation efficiency of the carbon particles has been shown to be between 89% and 95%. FT-IR spectra analysis indicates that the carboxylic groups were complexed with Fe^3+^ ions, and the carbon particles were successfully encapsulated within the gellan-based hydrogel particles.

The surface morphology of the particles was examined using scanning electron microscopy, revealing that the gellan particles are spherical with a smooth surface for the samples without carbon particles encapsulated, which becomes more compact as the cross-linking degree increases. The hydrogel particles were characterized physico-chemically, and their stability and swelling degree values were measured. The results showed that the particles are stable, with transmittance values between 97.8% and 99.2%, after maintaining them for 10 days in double-distilled water. The swelling degree of the particles with and without encapsulated carbon particles increases with the decrease of the cross-linking degree. The swelling degree value decreases when the polymer concentration increases.

The results demonstrate that the obtained polymer composites are effective adsorbents for the uptake of Pb^2+^ and diclofenac from an aqueous solution and can be used in at least four cycles of sorption/desorption. Further, the sorption capacity of polymer composites in relation to Pb(II) ions can be augmented significantly by embedding highly activated carbon structures.

Future research will address development of hydrogel particles cross-linked with other transitive metal ions in order to obtain matrices with lower reticulation degree. Also, new monomers are targeted with the aim to improve the porosity of hydrogels.

## 4. Materials and Methods

### 4.1. Materials

Deacetylated Gellan Kelkogel with a molecular mass of Mw = 2.351 × 10^5^ was used. The molecular mass was determined at 25 °C using the “automatic continuous mixing” method developed by Reed et al. [81]. FeCl_3_ × 6 H_2_O and NaCl were purchased from Sigma Aldrich, Burlington, MA, USA.

Our group obtained Carbon micro-nanostructures (CNMS) in the lab according to a protocol described in [82]. Our prior work has also reported the morpho-structural characteristics of the synthesized carbon particles performed through EDX, FT-IR, Raman, DLS, XRD, and HR-SEM [82].

Diclofenac—a non-steroidal anti-inflammatory drug—can be found especially in wastewater discharged by the pharmaceutical industry, a well-known endocrine disruptor contaminant due to its widespread use.

### 4.2. Methods

#### 4.2.1. Preparation of Hydrogel Microparticles Cross-Linked with FeCl_3_ × 6 H_2_O with or Without Carbon Particles Encapsulated

In order to obtain gellan particles that are ionically cross-linked with ferric chloride hexahydrate, first, gellan was dispersed in 10 mL of 0.05 M NaCl solution to obtain final concentrations of 0.5%, 1%, and 1.5%, respectively. The mixture was stirred at 300 rpm and heated to 80–90 °C until the gellan was completely dissolved. Afterward, the polymer solution was cooled to 45 °C and extruded into drops using an automatic pipette, which were then placed in a cross-linking bath containing solutions with different concentrations of FeCl_3_ × 6 H_2_O (10 mM, 20 mM, and 30 mM). The gellan particles were left in the solution at 4 °C for 12 h for stabilization, after which they were washed three times with double-distilled water to remove excess FeCl_3_ × 6 H_2_O. Finally, the particles were stored in well-closed containers in the refrigerator until further characterization.

Different amounts of carbon particles were used (10 mg, 20 mg, and 30 mg) for encapsulation in gellan hydrogel. These were added to the gellan solution after the polysaccharide had completely dissolved and then stirred at 65 °C and 500 rpm for 6 h to get a suspension of carbon microparticles in the gellan solution. The suspension was extruded using an automatic pipette into a cross-linking bath with a volume of 25 mL. The cross-linking bath contained solutions with different concentrations of FeCl_3_ × 6 H_2_O—10 mM, 20 mM, and 30 mM. These composite microparticles are maintained in the cross-linking bath for 12 h at a temperature of 4 °C. Afterward, the excess FeCl_3_ is removed by washing them with double-distilled water. Finally, the microparticles were kept in closed containers in a refrigerator at a temperature of 4–6 °C until subsequent testing.

#### 4.2.2. Encapsulation Efficiency of Carbon Microparticles

The encapsulation efficiency (Ef%) of carbon particles in gellan hydrogel was determined by measuring the amount of carbon particles left in the preparation recipient after extrusion. In order to separate the material from the gellan solution, we used syringe filters of polyethersulfone from ISOLAB with a pore diameter of 100 nm, separately, for each sample. Each syringe filter was weighed before the experiment started. Because the gellan solution with CNMS from the recipient’s walls was very viscous, 14 mL of bidistilled water was added, and it was heated at 80 °C to dilute the solution and detach the CNMS from the physical gel formed. Once the solution cooled to 45 °C), it was centrifuged at 6000 rpm for 30 min, and the CNMS settled at the bottom of the centrifuge tube. The bidistilled water without the CNMS was then removed and replaced with a fresh amount of up to 14 mL. The centrifugation process was repeated three times to ensure the removal of gellan gel. The CNMS suspension obtained after the last centrifugation was vortexed and filtered through pre-weighed syringe filters. The CNMS was trapped inside the filters; after drying them at 50 °C), the filters’ weight was measured until their weight remained constant. The immobilization efficiency was determined with Equation (2):

(2) $$E_{i}=\frac{m_{Ei}-m_{Es}}{m_{Ei}}\times 100$$

The m_Ei_ is the amount of initial CNMS added for encapsulation, and the m_Es_ is the amount of CNMS found in the recipient that was not immobilized.

#### 4.2.3. FT-IR Spectroscopy

FT-IR spectra were recorded using a Shimadzu IR Affinity 1S spectrometer in the 400–4000 cm^−1^ range via the KBr method for gellan, hydrogel particles without (SM22), and with carbon particles immobilized (SC22-2).

#### 4.2.4. Scanning Electron Microscopy

Scanning electron microscopy was used to characterize particles, with or without carbon particles encapsulated, to determine their surface morphology. The particles were dried, metalized with gold using a sputter deposition device, and analyzed using a Hitachi SU 1510 electron microscope (Hitachi Company, Tokyo, Japan).

#### 4.2.5. Stability in Water of Gellan-Based Hydrogel Microparticles

The study aimed to determine the stability of polymeric composites in bidistilled water. The less stable particles are believed to release polymer fragments, which can cause the aqueous solutions to become turbid. Also, the carbon particles may diffuse from the polymer matrix into the surrounding environment, which can also affect the turbidity of the solution.

The transmittance T% is measured using a Boeco S-20 spectrophotometer (Boeckel, Hamburg, Germany) at a wavelength of 550 nm for turbidity evaluation. This measurement is done both in the extrusion solution and in bidistilled water. The obtained FeCl_3_ ionically cross-linked gellan particles (1 g) were kept in bidistilled water (20 mL) for 10 days. Transmittance for each sample was measured at different time intervals in triplicate. Table 2 shows the average values of the results obtained ± StDev.

#### 4.2.6. Swelling Degree

The swelling degree (Q%) was assessed for the obtained hydrogel particles and composites. The swelling degree was determined gravimetrically by evaluating the ratio of water retained by the particles to their weight when dry. To obtain the mass of dry particles (M_dry_), samples with and without the encapsulated carbon particles were dried at 50 °C until their weight remained constant.

The dried samples were placed into 10 mL of a 0.05 M NaCl solution with a pH of 6.5. After different time intervals, the solution was removed using filtration, and the excess solution was blotted off the sample surfaces using filter paper. The weight of the swollen particles (M_swollen particles)_ was determined by repeatedly weighing the samples until their weight was constant. In order to determine the amount of water retained by the hydrogel particles, the weight of the swollen particles (M_swollen particles_) was subtracted from the weight of the dry particles (M_dry_), giving the amount of water retained (M_water_). The samples were then weighed and placed back into a 0.05 M NaCl solution. This process was repeated at predetermined time intervals until equilibrium was reached. The swelling degree was expressed as the ratio between the amount of water within the particles at each time interval and the amount of completely dry particles.

The degree of swelling was calculated for both types of particles (with or without carbon particles encapsulated), with the Equation (3):

(3) $$Q(\%)\;=\frac{M_{\mathrm{water}}}{M_{\mathrm{dry}\;\mathrm{sample}}}\times \;100$$

where: M_water_—the weight of water remaining/absorbed in the samples (g)

M_dry sample_—the weight of the dry sample.

The degree of swelling was measured three times, and the results are given as average value ± STDEV.

The normalized degree of swelling (*α*) is the degree of swelling at time *t* (*Q_t_*) divided by the degree of swelling at equilibrium (*Q_eq_*) [83]

(4) $$\alpha =\frac{Q_{t}}{Q_{eq}}$$

- Swelling degree kinetics

The swelling kinetics study was conducted to analyze the swelling results and characterize the structure of the composite hydrogel particles. Different mathematical models were used to calculate the swelling kinetics constants. The swelling kinetics are best explained by the mathematical model that exhibits the highest correlation coefficient (R^2^). In this study, the mathematical models used to analyze the swelling kinetics were:

(a) The model by Peppas [84] can be represented as

(5) $$\alpha ={k\times t}^{n}$$

*α* represents the normalized degree of swelling. The exponential factor *n* describes the type of transport mechanism. The hydrogel constant is denoted by *k*, and *t* represents the swelling time.

(b) The 1st order kinetics can be expressed as follows

(6) $$\alpha =\left(1-{A\times e}^{-kt}\right)$$

where *A* is the pre-exponential factor.

(c) The second-order kinetics can be expressed as follows

(7) tMt=1k×M∞2+1M∞×t

The mass of the hydrogel at equilibrium is denoted as *M*_∞_, while *M_t_* represents the mass of the hydrogel at time *t*.

The swelling kinetics of the hydrogels were investigated to fit the swelling behavior results to the Voight equation.

(8) α=1−e−tτ 

where *τ* is a parameter that represents the equilibrium swelling rate and can be found by graphing −ln(1 − *α*) against time. By calculating the slope of the resulting straight line, *τ* can be determined (slope = 1/*τ*).

#### 4.2.7. Atomic Adsorption and UV-VIS Spectroscopies

Atomic adsorption spectroscopy (AAS 932 GBC Scientific Equipment, Braeside, Victoria, Australia) was used to assess the concentration of metal ions. Dilutions were made to obtain values that fell within the linear ranges. The pharmaceutical model pollutant, diclofenac molecule, was determined using a UV-VIS Hitachi 5100 Spectrophotometer (Hitachi High-Tech Corporation, Hitachinaka, Japan) (λmax = 275 nm). Before analysis, calibration curves were performed, and the minimum determination coefficient admitted R^2^ > 0.995. The samples were filtered using cellulose acetate membrane filters (ISOLAB, Eschau, Germany) (45 μm porosity).

Batch sorption tests were carried out at constant temperature (25 ± 1 °C) in Erlenmeyer flasks of 25 mL of Pb^2+^ solution (20 mg/L) under stirring. In case of diclofenac adsorption tests, the initial concentration was 100 mg/L, and the batch tests were conducted in 100 mL volume flasks. Spent adsorbents were also regenerated using a 3 M KCl solution. Equilibrium time for both, sorption and desorption steps, respectively was 1440 min. All sorption tests were performed in duplicate.

#### 4.2.8. Statistical Analysis

A 2-tailed Student T-test was used to perform statistical analysis. A value of *p* < 0.05 was considered significant. All the experiments are performed in triplicate, and the results are given as average value ± standard deviation.

## Figures and Tables

**Table 1:** Experimental design for hydrogel particles cross-linked with FeCl_3_ with CNMS.

Nr. Crt.	Sample Code	[Gellan] (%)	[FeCl_3_ × 6 H_2_O] (mM)	[CNMS] (mg)
1	SM1	1	10	0
2	SM2-1	0.5	20	0
3	SM2-2	1	20	0
4	SM2-3	1.5	20	0
5	SM3	1	30	0
6	SC11	1	10	10
7	SC12	1	20	10
8	SC1-3	1	30	10
9	SM2-1	0.5	20	0
10	SC22-1	0.5	20	20
11	SC22-2	1	20	20
12	SC22-3	1.5	20	20
13	SC2-3	1	30	20
14	SC31	1	10	30
15	SC3-2	1	20	30
16	SC33	1	30	30

**Table 2:** Transmittance values for FeCl_3_ cross-linked hydrogel particles with and without encapsulated carbon particles. The values were determined after different storage periods in aqueous media for 248 h.

Samples	Transmittance T %				
	In double Distilled Water				
	12 h	72 h	148 h	196 h	248 h
SM1	99.6 ± 0.15	99.4 ± 0.05	99.4 ± 0.05	99.3 ± 0.05	99
SM2-2	99.2	99 ± 0.05	99 ± 0.05	98.8 ± 0.05	98.6 ± 0.05
SM3	99.25 ± 0.05	98.45 ± 0.05	98.3	98 ± 0.05	98 ± 0.05
SM2-1	99.2	99.1 ± 0.1	99.1 ± 0.05	99.1 ± 0.05	99 ± 0.1
SM2-3	99.7 ± 0.1	98.8 ± 0.05	98.8 ± 0.05	98.7	98.6 ± 0.1
SC22-1	99.1 ± 0.1	98.8 ± 0.1	98.7 ± 0.05	98.6	98.5 ± 0.05
SC22-3	99.6 ± 0.2	98.8 ± 0.1	98.8 ± 0.05	98.6 ± 0.05	98.4 ± 0.05
SC1-1	99.8 ± 0.05	99.3 ± 0.25	99.2 ± 0.15	99.2 ± 0.15	99.2 ± 0.15
SC1-2	100	99.6 ± 0.05	99.4 ± 0.05	99.1 ± 0.15	99.1 ± 0.15
SC1-3	100	99.3 ± 0.05	99.1 ± 0.05	99 ± 0.1	99 ± 0.1
SC2-1	100	99.4 ± 0.1	99.4 ± 0.1	99.3 ± 0.05	99.3
SC22-2	100	99.5 ± 0.1	99	98.7	98.7
SC2-3	100	99.3 ± 0.05	99.2 ± 0.1	99 ± 0.2	98.9 ± 0.05
SC3-1	100	99.5 ± 0.05	99.2 ± 0.1	99.1 ± 0.05	98.9 ± 0.1
SC3-2	100	99.2	99.1 ± 0.1	99.1 ± 0.2	99 ± 0.15
SC3-3	100	98.3 ± 0.15	98.2 ± 0.2	97.9 ± 0.15	97.8 ± 0.1

**Table 3:** The *n*, *k*, and *τ* values were obtained after integrating the swelling data obtained in different kinetic diffusion models.

Proba	Qmax	Peppas				First Order				Second Order	
		*n*	*k*	*r^2^*	*K* × 10^3^ (min)	*k* (h)	*τ* (min)	*τ* (h)	*r* ^2^	*k*	*r* ^2^
SM1	1076.97	0.22	0.22	0.948	2.8	0.169	357.14	5.9	0.952	0.148	0.955
SM2-1	1089.65	0.17	0.35	0.909	8.2	0.441	121.95	2.27	0.965	0.09	0.999
SM2-2	794.79	0.18	0.26	0.941	2.2	0.132	454.55	7.56	0.825	0.12	0.994
SM2-3	437.14	0.27	0.14	0.945	2.2	0.131	454.55	7.61	0.808	0.218	0.981
SM3	735.79	0.13	0.38	0.959	1.8	0.11	555.56	9.07	0.913	0.132	0.996
SC1-1	930.99	0.09	0.56	0.966	5.6	0.336	178.57	2.97	0.973	0.106	0.999
SC1-2	771.49	0.2	0.3	0.989	8.6	0.516	116.28	1.94	0.94	0.128	0.999
SC1-3	724.39	0.14	0.4	0.878	5.3	0.366	163.93	2.73	0.945	0.136	0.999
SC2-1	818.5	0.16	0.39	0.95	8.0	0.478	125	2.09	0.889	0.121	0.999
SC2-2	762.67	0.19	0.3	0.988	5.8	0.35	172.41	2.85	0.978	0.129	0.999
SC2-3	716.91	0.26	0.19	0.971	5.2	0.31	192.31	3.22	0.976	0.136	0.999
SC3-1	707.07	0.11	0.51	0.972	5.2	0.314	192.31	3.18	0.926	0.14	0.999
SC3-2	667	0.15	0.39	0.923	4.8	0.286	208.33	3.49	0.956	0.148	0.999
SC3-3	548.64	0.12	0.44	0.98	4.0	0.243	250	4.12	0.891	0.18	0.998
SC22-1	950.72	0.35	0.12	0.931	8.6	0.513	116.28	1.95	0.93	0.1	0.992
SC22-3	334.29	0.26	0.2	0.958	4.6	0.278	217.39	3.6	0.906	0.294	0.999

**Table 4:** Shows several results reported in literature concerning lead ions and diclofenac removal, respectively, on various synthesized polymeric composites.

Nr. Crt.	Adsorbent	Contaminant	Initial Concentration (mg/L)	Adsorption Capacity (mg/g)	Reference
1	Cellulose-based adsorbent	Pb^2+^	2000	810	[71]
2	Carboxymethyl cellulose	Pb^2+^	500	110	[72]
3	Chitosan-maleic anhydride/ Polyvinyl alcohol/ Silk fibroin	Pb^2+^	1000	168.9	[73]
4	Magnetic-chitosan/ Cellulose-microspheres	Pb^2+^	120	45.8	[74]
5	Carboxymethylated-bacterial cellulose	Pb^2+^	100	60.4	[75]
6	SC11	Diclofenac	5	4.1	[76]
7	SC12	Diclofenac	400	165.5	[77]
8	SC1-3	Diclofenac	16	23.3	[78]
9	SM2-1	Diclofenac	800	253.3	[79]
10	SC33	Diclofenac	1000	493.8	[80]

## Data Availability

Data are contained within the article.
